# Geographical Distribution of Exposure to Fires in the United States Between 2004 and 2023

**DOI:** 10.1029/2025GH001455

**Published:** 2026-11-09

**Authors:** Rand Sakka, Qiang Pu, Liora Sahar, Leticia M. Nogueira, Yang Liu

**Affiliations:** ^1^ Independent Scholar Gainesville FL USA; ^2^ Rollins School of Public Health Emory University Atlanta GA USA; ^3^ Department of Behavioral Science and Health Equity College for Public Health and Social Justice Saint Louis University St. Louis MO USA; ^4^ Geospatial Solutions Team American Cancer Society Atlanta GA USA; ^5^ Surveillance, Prevention, & Health Sciences Research American Cancer Society Atlanta GA USA

**Keywords:** wildfires, prescribed fires, fire exposure, climate change, environmental justice, environment, geography, emergency health

## Abstract

Fire activity is increasing across the United States. Proximity to fires poses health risks beyond smoke exposure including injury, mental health impacts, and contamination of water and soil. However, the predominance of smoke‐focused studies has contributed to public health strategies centered on mitigating smoke exposure. This study aims to characterize geographic patterns of population‐weighted fire burden to inform public health and fire management. Population‐weighted fire‐day burden (person days) were calculated for each census tract and year by multiplying the tract population by number of days with at least one satellite‐detected active fire. This metric captures the frequency of detected fire presence, including recurrent smaller fires, rather than smoke, fire intensity, or fire severity. These measures were summarized nationally, by region, and by demographic groups. Temporal trends between 2004 and 2023 were evaluated using regression models, and region‐specific percentiles identified census tracts with fire days in the 99th percentile every year (i.e., consistently high). Between 2004 and 2023, there were 9.12 billion population‐weighted fire‐days in the United States, 64% of which occurred in the South. Population‐weighted fire burden increased significantly over the study period nationally and in most census tracts. Census tracts with persistently high fire‐day burden were concentrated in Florida, consistent with regions where frequent agricultural burning occurs. Population‐weighted fire burden is disproportionately high among individuals residing in the South, highlighting the importance of tailoring strategies for mitigating the health risks of fires to local fire practices to protect the health and safety of residents in the region.

## Plain Language Summary

Fire activity is increasing in the United States with climate change. Exposure to fires presents various health risks beyond smoke exposure, including contamination of water and soil, injury, and mental health challenges. However, most studies focus on smoke exposure, causing public health agencies to prioritize smoke reduction strategies. Population‐weighted fire burden was calculated by multiplying satellite‐detected active fire days by the population of each census tract, then summarizing estimates annually at the national and regional levels, and by demographic groups. Census tracts with unusually high numbers of fire days, which may be especially important target for interventions, were identified by comparing each tract to regional patterns over time. During the study period, there were 9.1 billion person‐days of population‐weighted fire burden, the majority occurring in the South. Population‐weighted fire burden increased between 2004 and 2023, both nationally and in almost 90% of census tracts. Census tracts with consistently high fire activity were clustered in a predominantly agricultural region in Florida with hay and sugarcane production. Population‐weighted fire burden was highest among residents of the South, emphasizing the need for fire management and public health strategies to reduce potential health impacts.

## Key Points

- 64% of population‐weighted fire burden occurred in the South region
- Population‐weighted fire burden significantly increased nationally and in 89.7% of census tracts
- Population‐weighted fire burden appeared to increase more rapidly in the South region of the US based on available Hazard Mapping System data

## 1 Introduction

Fire activity, which is closely tied to temperature increases and drought, is increasing in the United States and poses major safety and public health risks (Schoennagel et al., 2017). Fires vary by size, intensity, fuel and ignition type, frequency, and seasonality. These characteristics define different fire regimes including wildfires and prescribed burning such as agricultural burns, and cultural burns. Fire risks go beyond the health consequences associated with exposure to air contaminated with fire smoke and include physical and mental health risks associated with injury, displacement (Suresh et al., 2024), property loss, erosion of social networks (Riley et al., 2024), and exposure to contaminated water and soil (Alexakis, 2020; Taparra et al., 2023; Xu et al., 2020). High‐intensity fires, such as wildfires, can physically alter water bodies by burning vegetation that typically intercepts waterflow, increasing runoff and the risk of overflow. These fires can also chemically alter water bodies by increasing nitrogen and phosphorus which can trigger algal blooms (Paul et al., 2022). Even low‐intensity fires can alter soil chemical composition by decreasing dissolved organic carbon concentrations (Rebiffé et al., 2025).

Nonetheless, previous national studies have prioritized smoke related exposure (Vargo et al., 2023), rendering other fire‐related hazards less visible and therefore harder to address. While fire regimes in the United States are diverse, varying by size, intensity, fuel and ignition type, frequency, and seasonality, national assessments of geographical patterns of fire hazards have focused solely on large fires (Rad et al., 2023). Predilections for focusing on fire elements that have high visibility (e.g., large fires, fire smoke) restricts media attention, scientific efforts, and public resources to regions where large fire events and expansive smoke plumes are prevalent, such as the West region of the United States (Environmental Protection Agency, 2024; National Academies of Sciences, 2024). This framing obscures the smaller but more frequent fires that characterize fire regimes in the southeastern United States, and fires of all sizes present health hazards to nearby communities in all regions (Romanello et al., 2022), including small, prescribed fires (Afrin & Garcia‐Menendez, 2021). Some of these health hazards include premature mortality associated with wildland fire smoke (including both wildfires and prescribed fires) (Fann et al., 2018), as well as increased hospital admissions for asthma linked to agricultural burning, a form of prescribed fire (Arbex et al., 2007). Therefore, national estimates based on tract‐level fire‐day occurrence can better capture population‐weighted fire burden across diverse fire regimes, including fires of all sizes, intensities, and frequencies, and can inform the implementation of targeted and locally relevant strategies.

To date, public health agencies have focused on reactive strategies that prioritize reducing exposure to smoke released from fires (Stone et al., 2019), such as staying in indoor environments with good insulation and high‐efficiency heating, ventilation, and air‐conditioning (HVAC) filters, avoiding time spent outdoors, and evacuating, all of which can be challenging among individuals and communities with limited access to resources (Vargo et al., 2023). However, even when smoke is directed away from populations, communities may still be affected through changes in water quality, soil conditions, and other environmental effects (Ngole‐Jeme, 2019; Robinne et al., 2021), providing a rationale for using presence of local fire activity as a complementary indicator of exposure to fire related hazards in the present study. Importantly, 84% of wildfires are human‐caused and therefore preventable (Balch et al., 2017). Existing fire prevention strategies include targeted educational campaigns, such as the “firewise” and “think before you burn,” which train residents on measures that can be implemented to prevent fires, financial assistance for implementation of wildland best management practices, and environmental restoration (Balch et al., 2017).

Moreover, some communities are disproportionately exposed to environmental hazards, a fundamental environmental justice issue (Nogueira & Yabroff, 2024; Yamane & Baumhofer‐Merritt, 2024). Efforts to reduce exposure in these communities (i.e., prevention) are especially relevant for advancing public health goals (Kapadia, 2023), especially for environmental hazards such as fires, which can impact both global climate change and populations residing far from areas where fires burn (Gochfeld & Burger, 2011). Therefore, understanding geographical patterns of population‐weighted fire burden from fires of all sizes is crucial for implementing targeted strategies that go beyond reactive response to fire smoke and instead encompass fire prevention, which are especially relevant as fire activity increases in the era of climate change.

The goal of the present study is to characterize geographical distribution of population‐weighted fire burden, including fires of all sizes throughout the United States to provide a complementary indicator of potential local fire hazard presence that can inform the development and implementation of fire management and public health strategies. In this study, population exposure to fire activity is defined as a population measure of the presence of at least one satellite‐detected fire within a census tract on a given day (i.e., fire days). This metric serves as a complementary indicator of the potential presence of local fire‐related hazards, rather than a direct measure of individual‐level exposure, proximity to fire, fire intensity, or specific downstream health impacts.

## 2 Methods

To study geographic distribution of population‐weighted fire burden in the United States, we utilized a combination of satellite‐derived fire data and population data from the United States Census. The satellite‐derived fire data provided comprehensive and temporally consistent information on the geographical distribution and frequency of active fires across the United States. We obtained the satellite‐derived fire product from National Oceanic and Atmospheric Administration's (NOAA) Hazard Mapping System (HMS), covering 1 January 2004 to 31 December 2023 (NOAA, 2023). The HMS system integrates satellite imagery data from polar and geostationary satellites and image processing techniques such as automated fire detections and expert image analysis to produce fire and smoke products. Our study relies solely on finalized and quality‐controlled HMS active fire data, which includes coordinates, time stamps, and fire detection methods. The active fire data is derived from observations collected by four satellite instruments (AVHRR, MODIS, VIIRS, ABI) aboard 15 satellites in either sun‐synchronous or geostationary orbit. At present, HMS fire detection primarily relies on VIIRS and ABI while MODIS and AVHRR were used historically and are no longer operational within the HMS system (National Oceanic and Atmospheric Administration, 2026). Fire detection depends on the spatial resolution of instruments, with coarser data requiring larger or more intense fires for detection. Current operational fire detection algorithms, such as those used in MODIS, VIIRS, and ABI, can detect fires covering at least 0.01% of a pixel (≥100 m^2^ for a 1 km × 1 km pixel) at temperatures ≥800 K. To improve accuracy, expert image analysis is applied to HMS fire product to account for potential commission and omission errors of fire detection. Commission errors (false positives) occur when bright surfaces, such as fresh burn scars, sandy soils, or sun glint, are misclassified as active fires. Conversely, omission errors may result from factors that obstruct fire detection, such as terrain, forest canopies, cloud cover, and land/water misclassification, leading to missed fire events.

Our population data, including total and race‐specific population counts, were derived from the decennial census data of 2000, 2010, and 2020 and were harmonized to 2010 census tract boundaries. We used the geographic crosswalks data from the National Historical Geographic Information System (NHGIS) of the Integrated Public Use Microdata Series (IPUMS) (IPUMS, 2020; Schroeder et al., 2025). Population redistribution across incompatible census tracts was achieved by areal interpolation, which assumed that characteristics within a source zone (2000 and 2020 Census) are distributed in proportion to the population densities of the target zone (2010 Census) (Schroeder, 2007). However, this approach may have introduced measurement uncertainty in areas characterized by substantial within unit heterogeneity.

We then used decennial United States Census Bureau population data for years 2000, 2010, and 2020 at the census tract level to estimate annual intercensal population count and subgroups for the years 2004–2009 and 2011–2019 (United States Census Bureau, 2012) using the interpolation method given below:

(1) $$P_{t}=\left[P_{3652}\;*\;\left(t/3652\right)\right]+\left[P_{0}\;*\;\left(\left(3652\;–\;t\right)/3652\right)\right]$$

where *P*_*t*_ = population estimate for year *t*, *P*_3652_ = population count at the end of the interval, *t* = number of days elapsed since the start of the interval (366 added in for leap years), *P*_*0*_ = population at the beginning of the interval. The estimates of 2004–2020 population data are then used to fit a stepwise autoregressive time‐series model to forecast population counts for 2021–2023 separately for each census tract and demographic subgroups (SAS PROC FORECAST, method = STEPAR). STEPAR first detrends the data with a time‐trend model via ordinary least squares, then it fits an autoregressive model to the resulting residuals to identify significant lags (SAS Institute Inc., 2015). The time‐trend component is:

(2) $$P_{t}=b_{0}+b_{1}t+b_{2}t^{2}+u_{t}$$

where *P*_*t =*_ population in year *t* (a sequential integer from 1 to 20 corresponding to years from 2004 to 2023, model fitting years 2004–2020 for forecast years 2021–2023), *b*_*0*_ *=* intercept, *b*_*1*_ = linear trend coefficient, *b*_*2*_ = quadratic trend coefficient, *t =* time index, and *u*_*t*_ *=* residual at time *t*, which is modeled using an autoregressive process:

(3) $$u_{t}=a_{1}u_{t-1}+a_{2}u_{t-2}+\text{…}+a_{p}u_{t-p}+\varepsilon_{t}$$

Where *p* = number of lags retained after backward stepwise selection by STEPAR, *u*_*t*_ = residual at time *t*, *u*_*t*‐1_ *, u*_*t*‐2_ *,...u*_*t‐p*_ = lagged residuals at *t*‐1, *t*‐2, … *t*‐*p* from Equation 2, *a*_1_, *a*_2_, …, *a*_*p*_ are autoregressive coefficients, and *ε*_*t*_ = white‐noise error term a time *t* (SAS Institute Inc, 2015). The number of autoregressive lags *p* was selected automatically by the STEPAR procedure based on the data. The same forecasting approach was applied to the total population and each demographic subgroup. Population forecasts for 2021–2023 are inherently less certain than the interpolated estimates for earlier years, as interpolation uses two known decennial census counts while forecasting projects forward from 2020 without a subsequent census count to constrain the estimates. We linked HMS fire data with population data by overlaying fire coordinates onto census tract boundaries. Each fire event was assigned to the tract in which it occurred. If a fire fell on a boundary, it was allocated to the tract with the shortest distance to its centroid. Given that active fire locations may overcount actual fire events, we summarized fire occurrences using fire days, and treated a fire event as the presence of at least one detected fire within a census tract on a given day. Population‐weighted fire burden (person‐days) was then calculated by multiplying the number of fire days by the population of each census tract each year. Population‐weighted fire burden was compared between South, West, Midwest, and Northeast geographic regions and the 5 different sociodemographic groups: non‐Hispanic American Indian Alaska Native, non‐Hispanic Asian Pacific Islander, non‐Hispanic Black, Hispanic, and non‐Hispanic White as defined by the United States Census Bureau. (United States Census Bureau, 2022) Temporal changes in population‐weighted fire burden were evaluated by fitting mixed‐effects models estimated using maximum likelihood with tract‐specific random intercepts to account for repeated measures over time. Let *Y*_*it*_ denote person days of potential fire exposure in census tract *i* in year *t*, and *u*_*0i*_ denote the tract‐specific random intercepts, and *ε*_*it*_ as the error term. The unadjusted model estimating the average annual change in person‐days across all tracts was:

(4) $$Y_{it}=\beta_{0}+\beta_{\text{unadj}}^{t}t+u_{0i}+\varepsilon_{it}$$

Where *β*_unadj_ represents the average annual change in person‐days across all tracts and its statistical significance was evaluated using the Satterthwaite approximation for degrees of freedom. To decompose temporal changes in person‐days into components attributable to population dynamics versus changes in fire activity, we re‐estimated the model adjusting for *P*_*it*_, total population in census tract *i* in year *t*, using the following equation:

(5) $$Y_{it}=\beta_{0}+\beta_{\text{adj}}^{t}t+\beta_{2}\;\cdot \;P+u_{0i}+\varepsilon_{it}$$

Where *β*_*2*_ is the fixed effect of tract‐year population on fire person‐days, and *β* ^*t*^_adj_ represents annual change in person‐day adjusted for population. The proportion of the estimated temporal trend statistically accounted for by tract level population change (rather than fire activity) was calculated as the proportional reduction in the year coefficient after adjustment as follows:

(6) $$\left(\beta_{\text{unadj}}^{t}-\beta_{\text{adj}}^{t}\right)\;/\;\beta_{\text{unadj}}^{t}$$

Given the heterogeneous geographic distribution of fire activity across the United States, the distribution of population‐weighted fire burden was right‐skewed, and model residuals showed heteroscedasticity. To assess whether this violation affected the study's conclusions, a sensitivity analysis was conducted using log‐transformed population‐weighted fire burden as the outcome. This analysis yielded qualitatively consistent results, suggesting that the primary conclusions are robust to the skewed outcome distribution.

Finally, to identify census tracts with consistently high fire activity, we calculated region‐specific percentiles, and census tracts in the top 1% (99th percentile) were classified as high potential exposure in any given year. We then quantified the number of years in which each tract ranked in the top 1% within its region. We defined “consistently high” based on consistent occurrence over time, which are especially relevant for fire prevention strategies (instead of abnormal fire activity in a single year). Land cover characteristics in census tracts with consistently high fire activity were derived using the National Land Cover Database (NLCD). Census tract geometries were overlaid with NLCD raster data, and the proportion of each land cover type in each tract was calculated using area‐weighted extraction. To further characterize land use in tracts with consistently high fire activity, crops produced in these tracts were identified using USDA Cropland Data Layer (CDL), and a raster‐to‐polygon approach was implemented using area‐weighted extraction (i.e., cell value multiplied by coverage fraction) to estimate the proportion of each identified crop within each census tract boundaries. All study analyses were conducted using SAS 9.4, and figures were generated using R.

## 3 Results

United States residents experienced a total of 9.12 billion person‐days of population‐weighted fire burden between 2004 and 2023, with an average of 456 million person‐days per year (Figure 1a, Table S1). This total reflects burden from all satellite‐detected fires, including prescribed fires such as agricultural burns and wildfires of all sizes and intensities, as fire type distinctions cannot be made using AVHRR, MODIS, VIIRS, or ABI detections. The majority of population‐weighted fire burden (64%) occurred in the South, followed by 19% in the Midwest, 16% in the West, and 1% in the Northeast (Figure 1b).

**Figure 1:**
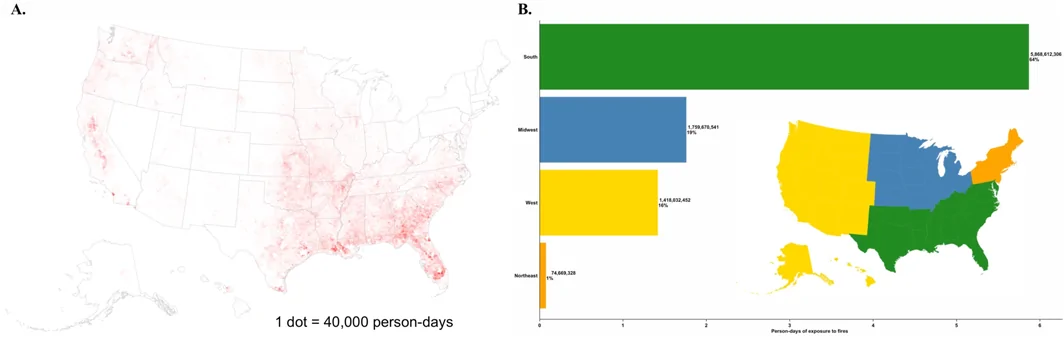
Geographic distribution of population‐weighted fire burden in the United States, 2004–2023. Notes: (a) Total person‐days of population‐weighted fire burden per United States census tract between 2004 and 2023. Each dot = 40,000 person‐days. (b) Total and percent person‐days of population‐weighted fire burden per geographic regions, as defined by the United States census.

Population‐weighted fire burden significantly increased during the study period at a rate of 19.94 million person‐days per year (*p* < 0.0001). In 2004 there was a total of 292 million person‐days of population‐weighted fire burden, compared to 755 million in 2023. For each one‐year increase, the average expected population‐weighted fire burden (*β^t^*_unadj_) increased by 274.12 across census tracts. After adjusting for tract‐level population changes, the expected fire day burden (*β* ^*t*^_adj_) increased by 227.42 per year. Meaning 17% of the estimated temporal trend in population‐weighted fire burden was statistically accounted for by tract level population change under the specified model. On a regional scale, changes in population in the Northeast contributed to 0.95% of the changes in person‐day with a *β^t^*_unadj_ value of 26.56 and a *β* ^*t*^_adj_ value of 26.3, 11.8% in the West (*β^t^*_unadj_: 219, *β* ^*t*^_adj_: 193.16), 2% in the Midwest (*β^t^*_unadj_: 212.26, *β* ^*t*^_adj_: 207.94), and 25.4% in the South (*β^t^*_unadj_: 475.67, *β* ^*t*^_adj_; 354.79). In 2010, there were 71,141 census tracts in all 50 states, with 31,343 (44%) census tracts experiencing a fire day at least once in the study period. Of these, 7,400 (23.6%) had statistically significant changes in person‐days of population‐weighted fire burden. Of these, 6,449 (87.1%) census tracts experienced positive and statistically significant changes, and 951 (12.9%) experienced significant decreases in person‐days of population‐weighted fire burden during the study period. The geographic distribution of these changes is shown in Figure 2a (Figure 2a). Person‐days of population‐weighted fire burden increased more rapidly in the South region of the United States than other regions (Figure 2b).

**Figure 2:**
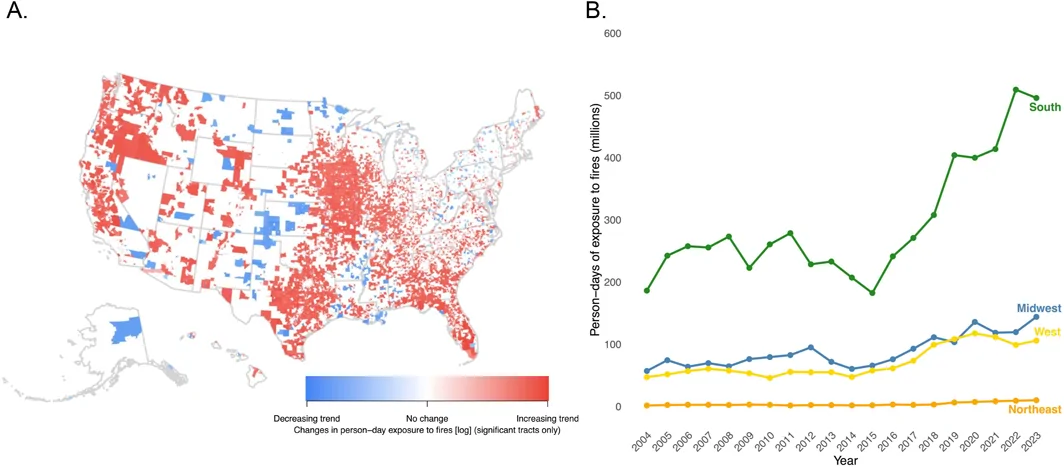
Changes in Person‐Days of population‐weighted fire burden in the United States, 2004–2023. Notes: (a) Changes in population‐weighted fire burden (person‐days) in the United States between 2004 and 2023. (b) Increases in population‐weighted fire burden (person‐days) by geographic regions in the United States.

The majority of total person‐days of population‐weighted fire burden was observed among United States residents who self‐identify as non‐Hispanic White (6.8 billion person‐days), the largest demographic group in the United States, followed by 1.1 billion person‐days among residents who self‐identify as non‐Hispanic Black, 955 million who self‐identify as Hispanic, 177 million person‐days among residents who self‐identify as American Indian or Alaskan Native, and 97 million person‐days among residents who self‐identify as Asian American or Pacific Islander (Table 1). However, individuals who self‐identify as American Indian or Alaskan Native were disproportionately burdened by fires, experiencing an average of 10.8 person‐days of population‐weighted fire burden per a 100,000 person‐days, compared to 4.6 person‐days per 100,000 person‐days among residents who self‐identify as non‐Hispanic White (Table 1).

**Table 1:** Population‐Weighted Fire Burden (Person Days) of by United States Demographic Characteristics

Demographic group	Percent of US population^a^	Total person days with fire activity	Person days with fire activity per 100,000 person‐days
American Indian or Alaskan Native	0.7	177,095,968	10.8
Asian American or Pacific Islander	5.3	97,338,909	0.7
Hispanic	16.5	954,859,441	2.3
Non‐Hispanic Black	15.4	1,146,480,670	3.5
Non‐Hispanic White	64.1	6,769,814,972	4.6

^a^ Estimates from 2010 United States Census Bureau available at https://www.census.gov/programs‐surveys/popest.html Denominator in the final column represents the total population‐time contributed by each demographic group over the 20‐year study period.

There were 703 census tracts with a number of fire days in the top 1% at least once during the study period, of which 9 had consistently high numbers of fire days (i.e., in the top 1% every year from 2004 to 2023). All census tracts with consistently high number of fire days were clustered near Lake Okeechobee in Florida, a state in the South region of the US (Figure 3a). The number of fires per year ranged from 61 to 248 (Figure 3b). Among census tracts with consistently high fire days, the dominant land cover type was planted/cultivated (Figure 3c), and the dominant crops were hay and sugarcane (Figure 3d).

**Figure 3:**
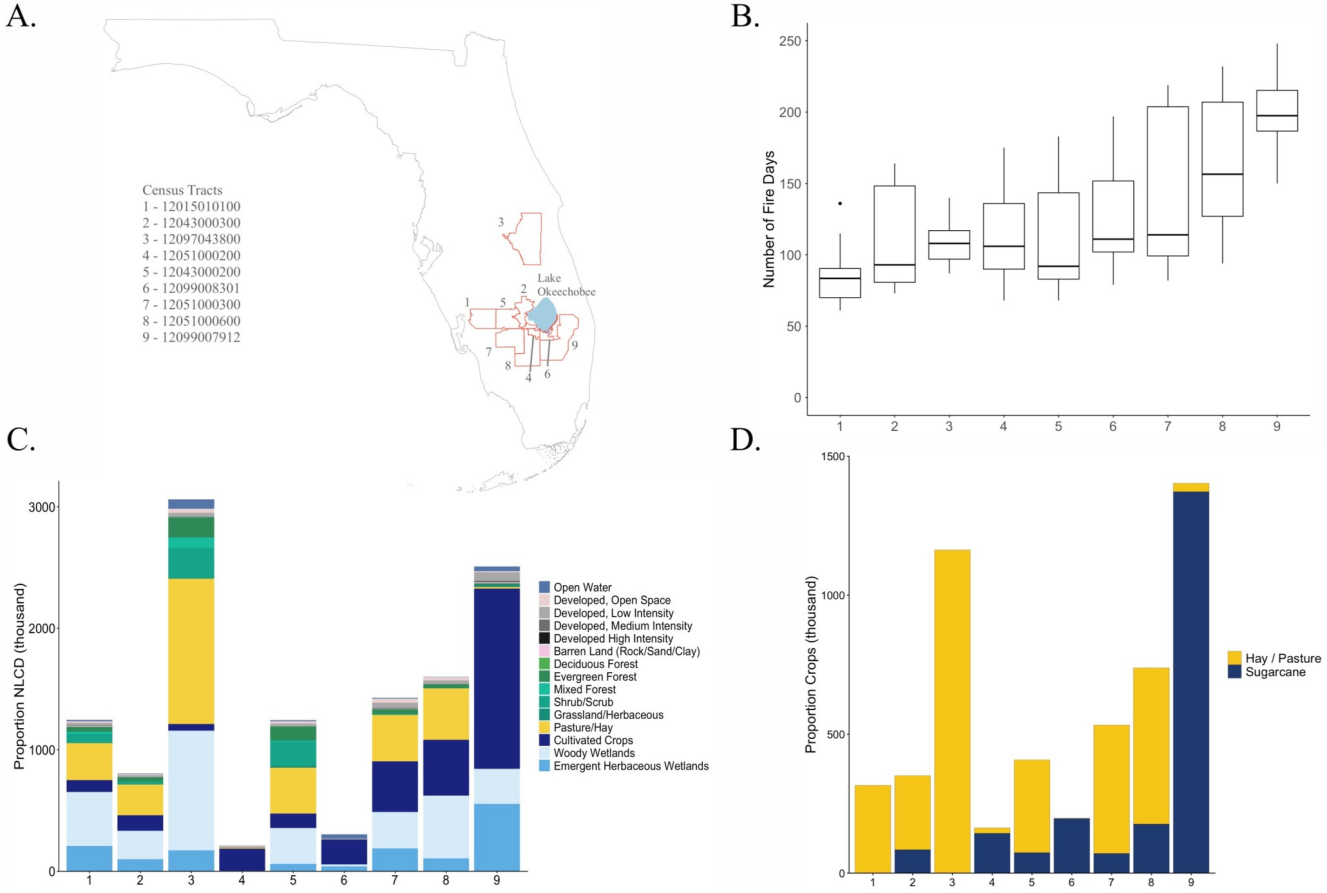
Census tracts with a consistently high number of days with fire activity in the United States, 2004–2023. Census tracts are marked 1–9 in the map and graphs and are identified by their 11‐digit numeric FIPS identifier. Notes. (a) Location of census tracts with a consistently high number of fire days (99th percentile) every year (2004–2023). (b) Distribution of number of fire days in each census tract each year. (c) Land cover type proportions within census tracts with a consistently high number of fire days. (d) Crop production within census tracts with a consistently high number of fire days.

## 4 Discussion

Population‐weighted fire burden impacted residents of all regions of the United States and has increased significantly in the past two decades. Although some of this increase may partly reflect improvements in HMS satellite detection capability over time, the observed increase represents a substantial public health burden. In our analysis, shifts in population distribution statistically accounted for 17% of the increase, suggesting that rising fire burden was associated with increases in detected fires. Geographic distribution of population‐weighted fire burden is uneven in the United States, with individuals residing in the South region (rather than the West) being more frequently burdened compared to other regions. Moreover, the South region is also where population‐weighted fire burden patterns are consistent with regions characterized by frequent prescribed fires, including agricultural burns, as documented in prior literature (Melvin, 2020; Nowell et al., 2022) and where historical population‐weighted fire burden may have been underestimated by satellite‐based fire detection systems. Therefore, our findings provide a descriptive foundation that may inform the development of strategies for mitigating the health hazards of fires to local fire practices and fire regimes extending beyond the wildfire‐focused preparedness and response frameworks developed for the West region, where most studies have been focused (Childs et al., 2022; Huff et al., 2015; Jones & Berrens, 2017; Liu & Peng, 2019; Masri et al., 2021). Our findings of more frequent population weighted fire burden in the South align with a previous study that evaluated hazards associated with exposure to smoke from wildland fires, which include both wildfires and prescribed fires, throughout the continental United States and found fire hazards are not constrained to western states (O'Dell et al., 2021) and the largest health impacts occur in the South (Fann et al., 2018; Larsen et al., 2018; Rappold et al., 2017). In contrast to approaches that rely solely on smoke‐based metrics (Liu & Peng, 2019; Rad et al., 2023; Reid et al., 2016) our study provides a complementary indicator of potential local fire‐related hazard that may capture population‐weighted fire burden beyond what smoke‐based metrics alone can detect across different types of fires (Kizer, 2020) and fire regimes throughout the United States.

Fire regimes differ vastly across the United States; the West faces large and destructive wildfires, while the South has an established culture of frequent prescribed burning (Melvin, 2020). Prescribed fires are defined as planned wildland fires conducted under controlled conditions that are used for various land management objectives, including mitigation of hazardous wildfire risk, restoration of fire‐dependent ecosystems, and agriculture (Hiers et al., 2020; Kobziar et al., 2015) and also pose significant health hazards to nearby communities, especially in the southern United States (Afrin & Garcia‐Menendez, 2021; Maji et al., 2024; Nowell et al., 2022).

Due to policies and practices influencing how burn authorizations for prescribed fires are operationalized (Coleman, 2022) the health hazards posed by prescribed fires in the South disproportionately impact historically marginalized communities (Afrin & Garcia‐Menendez, 2021; Nowell et al., 2022). For example, in areas directly south of Lake Okeechobee, South Florida, authorizations for sugarcane burning incorporated plume‐dispersion modeling to prevent smoke from drifting into designated “sensitive” (i.e., affluent) areas, which consequently increases exposure in disenfranchised communities located closer to the agricultural fields (Coleman, 2022). Therefore, using population‐weighted fire burden as a metric is particularly relevant in regions where smoke‐centered measures are likely to underestimate fire‐related health hazards, including those posed by complex mixtures of volatile organic compounds and other short‐lived pollutants (Hartner et al., 2024; Manrique et al., 2025), and may obscure environmental justice implications for nearby communities.

Accordingly, in the present study, all census tracts with a consistently high number of fire days were located in the same region in Florida, near Lake Okeechobee, an area previously identified in the literature as having extremely high fire activity associated with sugarcane burning (a form of prescribed fire) (Nowell et al., 2022), with substantial environmental justice implications due to several documented health hazards associated with sugarcane burning (Hall et al., 2012). Importantly, burning is not required for sugarcane harvesting (Perillo et al., 2022), and has been banned in other countries, such as Brazil, providing a model for implementation of existing fire prevention strategies that have both public health and climate change mitigation co‐benefits (Valente & Laurini, 2021).

Our results of increasing population‐weighted fire burden align with previous studies, which also found the increase was not due to population shifts (Romanello et al., 2022). In the United States, the increase in fire frequency over time might be partly explained by growing adoption of wildfire mitigation strategies (i.e., small, prescribed burns) in the West (Melvin, 2020), where extreme wildfires are a major concern (Kizer, 2020). However, growing adoption of wildfire mitigation strategies does not explain the sharp increase in population‐weighted fire burden in the South, where there is no evidence of increasing number of prescribed fires (Cummins et al., 2023; Melvin, 2020; Nowell et al., 2018). Alternatively, improvement in HMS instruments' ability to detect small fires over time might have contributed to the statistically significant higher increase of population‐weighted fire burden in the South (Li et al., 2020), where small prescribed fires are common. Moreover, the use of multiple instruments contributing to HMS may have increased the likelihood of multiple detections of the same fire. However, the potential of multiple detections arising from the expansion of the instrument network is mitigated by our binary exposure definition (any fire vs. no fire). Consequently, multiple detections of the same fire by different instruments would not alter exposure classification once a fire was identified within the census tract‐year. Taken together, the limitations of HMS instruments in detecting small fires, especially in earlier years, combined with the lack of evidence of increasing frequency of small, prescribed fires in the South during the study period (Cummins et al., 2023; Melvin, 2020), indicates we might be underestimating population‐weighted fire burden in the region, especially in early years. Individuals who self‐identify as non‐Hispanic White were most frequently burdened by fires, while individuals who self‐identify as American Indian or Alaskan Native were disproportionately burdened in the United States. Therefore, implementation of fire prevention efforts in fire prone areas has tremendous potential for reducing fire burden in the entire population. Existing fire prevention strategies include environmental restoration, reducing accidental human‐caused ignitions (e.g., educating landowners on safe burning practices, preventing sparks from aging electrical infrastructure), and managing vegetation and accumulation of fuels. Managing vegetation and fuel accumulation can involve the use of prescribed fire, which is viewed as an effective fuel management tool (Kobziar et al., 2015). In turn, the use of prescribed fire can increase fire detections and short‐term exposure even while reducing the risk of large, high‐intensity wildfires. Additionally, small‐area prescribed fires conducted using Traditional Ecological Knowledge have been shown to be an important tool in fire management in the era of climate change (Roos et al., 2022).

While cultural burning practices probably contribute to some of the disproportionate fire burden among individuals who self‐identify as American Indian or Alaskan Native, future studies should investigate the role of other factors, such as post‐colonial agricultural and housing practices, which have reshaped landscape characteristics in the United States and might increase risk of involuntary exposure to fire in this population (Taparra et al., 2023). As the role of managed fire activity in maintaining the health of different ecosystems and preventing dangerous fires continues to grow (USFS, 2022), a better understanding of the public health implications of different burning practices will be crucial for developing effective preventive strategies.

Strengths of the study include the use of population‐weighted fire burden metrics, which provides a complementary approach to characterizing potential population exposure to fire hazards potentially missed by approaches that rely solely on smoke metrics. Additionally, leveraging spatially consistent fire detection from satellites, our nationwide study captures the distinct fire regimes in the country, providing insights for potential exposure prescribed fires given limitations of administrative records (i.e., fire permits). The study further considered all fire types, recognizing that both large, high‐intensity fires—common in the Western region of the United States—and small, frequent fires—common in the Southern region—pose public health hazards. Limitations of the study include population estimates for intercensal years, which might lead to over‐ or under‐estimation of population‐weighted fire burden at census tracts with rapidly growing or shrinking populations in some years, and/or exposure to hazards that are not adequately captured by proximity alone. Additionally, population estimates for 2021–2023 were based on forecasting rather than interpolation between known census counts and therefore carry greater uncertainty than estimates for earlier years. Any resulting error in tract‐level population forecasts may contribute to over‐ or under‐estimation of population‐weighted fire burden for those years. Another limitation is that some fires may have been undetected by satellites due to cloud coverage, possibly resulting in underestimation of population exposure to fires, especially in regions with frequent cloud coverage. Additionally, HMS is more sensitive to large fire activity and smoke plumes. The addition of higher resolution instruments used by HMS has improved fire detection capability over time (Li et al., 2020), which may partially explain the observed increases in fire frequency in the South. Because year was modeled as a continuous predictor in the trend analyses, improvements in detection sensitivity cannot be fully separated from true increases in fire occurrence. Notably, there is no evidence of an increase in frequency of prescribed fires in the South during the study period (Cummins et al., 2023; Melvin, 2020). In addition, the binary tract‐day indicator used in this study treats all fire days equivalently, regardless of the number of fires detected within a tract on a given day, fire size, intensity, duration, or its location within a tract. While this approach avoids overcounting multiple detections of the same event, future studies could build on this approach by incorporating fire characteristics to further differentiate levels of exposure within census tracts and near census tract borders. Finally, satellite data currently cannot be used to distinguish between different types of fires (e.g., wildfires, prescribed fires, agricultural burns) and all fire events were counted equally in the present study. Further research is needed to evaluate whether the health hazards associated with proximity to fire events vary by fire type, size, and/or intensity, as well as the effectiveness of different preventive strategies. In particular, future studies should also examine how small, prescribed fires, including frequent agricultural burning, impacts nearby air quality, including short lived pollutants, soil composition, and watersheds and waterbodies.

## 5 Conclusion

Population‐weighted fire burden was highest in the South region of the United States, where prescribed fire activity is consistent with frequent prescribed burning including agricultural burning, suggesting the value of tailored fire management strategies in areas beyond the West. There was an observed increase in population‐weighted fire burden in the United States between 2004 and 2023, particularly in the South. While population‐weighted fire burden was most frequent among individuals who self‐identify as non‐Hispanic White, it was disproportionately high among individuals who self‐identify as American Indian or Alaskan Native. Because fires present several public health and safety hazards that go beyond exposure to smoke, future studies should evaluate the different factors contributing to increasing fire burden in the United States population, especially in locations with consistently high numbers of fire days, such as South Florida, which may represent areas for future research and preventive efforts relevant to both public health and climate mitigation.

## Conflict of Interest

The authors declare no conflicts of interest relevant to this study.

## Supporting information

Table S1

## Data Availability

All data used for this study are publicly available. Data sets: The satellite‐derived fire data used for fire counts and person day exposure in the study were obtained from the National Oceanic and Atmospheric Administration's (NOAA) Hazard Mapping System (HMS) Fire and Smoke Product and are publicly available via [https://www.ospo.noaa.gov/products/land/hms.html#data]. Total population and demographic data for the years 2000, 2010, and 2020 were sourced from the Integrated Public Use Microdata Series (IPUMS) National Historical Geographic Information System (NHGIS) to obtain population counts and calculate person day exposure. Data is publicly available with a registration via [https://data2.nhgis.org/main] Satellite‐derived fire data merged with population data on the census tract level stored in Main_Dataset.csv accessible via [https://doi.org/10.5281/zenodo.17925232] (Pu et al., 2026) Land cover data were obtained from the National Land Cover Database (NLCD) Annual Land Cover product for 2021, covering the conterminous United States (CONUS), available through the Multi‐Resolution Land Characteristics (MRLC) Consortium [https://www.mrlc.gov/data] Crop type data were obtained from the USDA National Agricultural Statistics Service (NASS) Cropland Data Layer (CDL) for 2023, downloaded directly from the NASS CDL release page via [https://www.nass.usda.gov/Research_and_Science/Cropland/Release/index.php] The code used for estimating intercensal population counts, fire‐days of exposure, changes in population‐weighted fire burden through time, contribution of population shifts to changes in population‐weighted fire burden over time, and z‐scores is preserved at https://zenodo.org/records/21542818 (Sakka & Nogueira, 2026). Software: Version 9.4 of SAS Studio used for data cleaning, merging, and statistical analyses can be accessed via [https://www.sas.com/en_us/software/studio.html], available with a Proprietary commercial license. Version 2023.03.1 + 446 of RStudio used for creating the maps can be accessed via [https://posit.co/download/rstudio‐desktop/], open source.
