# Supplementary material for: Geographical Distribution of Exposure to Fires in the United States Between 2004 and 2023
Source: Geohealth. 2026 Nov 9;10(11):e2025GH001455. doi: 10.1029/2025GH001455 (PMC13746885; doi:10.1029/2025GH001455)
Supplement: Supplementary file 1 [file GH2-10-e2025GH001455-s001.docx]

*GeoHealth*

Supporting Information for

Geographical distribution of exposure to fires in the United States between 2004 and 2023

Rand Sakka, Qiang Pu^3,4^, Liora Sahar^2^, Leticia M. Nogueira^1^, Yang Liu^3^

^1^Surveillance, Prevention, & Health Sciences Research, American Cancer Society, Atlanta, Georgia, USA, ^2^Geospatial Solutions Team, American Cancer Society, Atlanta, Georgia, USA, ^3^Rollins School of Public Health, Emory University, Atlanta, Georgia, USA, ^4^Department of Behavioral Science and Health Equity, College for Public Health and Social Justice, Saint Louis University, St. Louis, Missouri, USA

**Contents of this file**

Table S1

**Introduction**

This file includes one supplementary table (Table S1) requested by Reviewer #1. This table summarizes total fire days, population, and total person-day exposure to fires by region and year. The total fire days reported in this table represent the number of fire days detected at the census tract level, which were then aggregated to the regional level.

| **Region** | **Year** | **Total Fire Days** | **Population** | **Population Changes** | **Total Person Day Exposure** | **Changes in Person Day Exposure** |
| --- | --- | --- | --- | --- | --- | --- |
| Midwest | 2004 | 16,681 | 11,450,347 |  | 56,982,299 |  |
| Midwest | 2005 | 21,304 | 13,187,677 | 1,737,330 | 74,269,060 | 17,286,761 |
| Midwest | 2006 | 18,448 | 12,589,094 | -598,583 | 63,801,933 | -10,467,127 |
| Midwest | 2007 | 20,004 | 13,317,994 | 728,900 | 69,524,906 | 5,722,973 |
| Midwest | 2008 | 18,621 | 12,594,939 | -723,055 | 64,441,360 | -5,083,546 |
| Midwest | 2009 | 21,712 | 14,099,709 | 1,504,770 | 76,092,420 | 11,651,060 |
| Midwest | 2010 | 22,895 | 14,895,752 | 796,043 | 79,250,700 | 3,158,280 |
| Midwest | 2011 | 24,566 | 13,210,138 | -1,685,614 | 82,503,933 | 3,253,233 |
| Midwest | 2012 | 27,666 | 15,035,521 | 1,825,383 | 94,892,506 | 12,388,573 |
| Midwest | 2013 | 20,727 | 13,527,689 | -1,507,832 | 71,648,235 | -23,244,271 |
| Midwest | 2014 | 17,233 | 12,176,347 | -1,351,342 | 60,291,646 | -11,356,589 |
| Midwest | 2015 | 19,481 | 12,624,132 | 447,785 | 65,663,954 | 5,372,308 |
| Midwest | 2016 | 21,732 | 13,459,946 | 835,814 | 75,728,558 | 10,064,604 |
| Midwest | 2017 | 26,147 | 15,332,841 | 1,872,895 | 92,681,525 | 16,952,967 |
| Midwest | 2018 | 30,448 | 17,423,647 | 2,090,806 | 111,180,783 | 18,499,258 |
| Midwest | 2019 | 27,943 | 18,469,190 | 1,045,543 | 103,171,618 | -8,009,165 |
| Midwest | 2020 | 38,312 | 18,808,477 | 339,287 | 135,740,942 | 32,569,324 |
| Midwest | 2021 | 32,673 | 18,674,403 | -134,074 | 118,404,139 | -17,336,803 |
| Midwest | 2022 | 32,347 | 18,575,882 | -98,521 | 119,440,549 | 1,036,410 |
| Midwest | 2023 | 38,246 | 20,126,568 | 1,550,686 | 143,959,476 | 24,518,927 |
| North | 2004 | 345 | 1,170,696 |  | 1,446,737 |  |
| North | 2005 | 520 | 1,755,073 | 584,377 | 2,203,729 | 756,992.00 |
| North | 2006 | 614 | 1,958,248 | 203,175 | 2,635,054 | 431,325 |
| North | 2007 | 581 | 1,693,755 | -264,493 | 2,515,369 | -119,685 |
| North | 2008 | 522 | 1,496,128 | -197,627 | 2,254,293 | -261,076 |
| North | 2009 | 647 | 1,970,755 | 474,627 | 2,846,198 | 591,905 |
| North | 2010 | 555 | 1,679,132 | -291,623 | 2,454,990 | -391,208 |
| North | 2011 | 324 | 1,107,076 | -572,056 | 1,463,899 | -991,091 |
| North | 2012 | 492 | 1,454,073 | 346,997 | 2,104,153 | 640,254 |
| North | 2013 | 465 | 1,546,293 | 92,220 | 2,061,723 | -42,430 |
| North | 2014 | 382 | 1,234,957 | -311,336 | 1,617,079 | -444,644 |
| North | 2015 | 400 | 1,406,490 | 171,533 | 1,811,054 | 193,975 |
| North | 2016 | 674 | 2,068,247 | 661,757 | 3,032,487 | 1,221,433 |
| North | 2017 | 537 | 1,846,408 | -221,839 | 2,420,322 | -612,165 |
| North | 2018 | 681 | 2,274,312 | 427,904 | 2,981,256 | 560,934 |
| North | 2019 | 1,370 | 3,597,857 | 1,323,545 | 6,258,688 | 3,277,432 |
| North | 2020 | 1,591 | 3,935,520 | 337,663 | 7,182,507 | 923,819 |
| North | 2021 | 1,835 | 4,442,017 | 506,497 | 8,334,128 | 1,151,621 |
| North | 2022 | 1,967 | 4,746,447 | 304,430 | 9,088,578 | 754,450 |
| North | 2023 | 2,109 | 4,772,888 | 26,441 | 9,957,084 | 868,506 |
| South | 2004 | 45,352 | 30,658,922 |  | 186,165,824 |  |
| South | 2005 | 57,705 | 34,065,845 | 3,406,923 | 242,295,020 | 56,129,196 |
| South | 2006 | 59,760 | 35,807,827 | 1,741,982 | 257,506,255 | 15,211,235 |
| South | 2007 | 60,239 | 35,253,269 | -554,558 | 255,447,439 | -2,058,816 |
| South | 2008 | 64,827 | 35,364,151 | 110,882 | 273,030,931 | 17,583,492 |
| South | 2009 | 52,198 | 32,380,913 | -2,983,238 | 222,713,766 | -50,317,165 |
| South | 2010 | 61,450 | 33,186,087 | 805,174 | 260,474,066 | 37,760,300 |
| South | 2011 | 65,436 | 33,241,985 | 55,898 | 278,532,714 | 18,058,648 |
| South | 2012 | 53,296 | 31,182,936 | -2,059,049 | 228,441,111 | -50,091,603 |
| South | 2013 | 54,376 | 31,871,088 | 688,152 | 232,859,091 | 4,417,980 |
| South | 2014 | 48,261 | 30,583,960 | -1,287,128 | 207,071,634 | -25,787,457 |
| South | 2015 | 42,333 | 29,043,584 | -1,540,376 | 182,310,656 | -24,760,978 |
| South | 2016 | 56,075 | 33,331,289 | 4,287,705 | 241,190,481 | 58,879,825 |
| South | 2017 | 61,803 | 35,172,978 | 1,841,689 | 270,806,012 | 29,615,531 |
| South | 2018 | 68,425 | 36,977,481 | 1,804,503 | 307,658,861 | 36,852,849 |
| South | 2019 | 87,419 | 40,606,264 | 3,628,783 | 403,827,662 | 96,168,801 |
| South | 2020 | 85,031 | 39,812,810 | -793,454 | 399,703,211 | -4,124,451 |
| South | 2021 | 86,694 | 41,509,906 | 1,697,096 | 413,653,378 | 13,950,167 |
| South | 2022 | 105,564 | 44,468,189 | 2,958,283 | 509,139,213 | 95,485,835 |
| South | 2023 | 101,627 | 45,466,323 | 998,134 | 495,784,983 | -13,354,230 |
| West | 2004 | 12,531 | 7,803,471 |  | 47,077,071 |  |
| West | 2005 | 13,579 | 8,287,784 | 484,313 | 51,546,602 | 4,469,531 |
| West | 2006 | 14,802 | 8,455,988 | 168,204 | 56,727,773 | 5,181,171 |
| West | 2007 | 15,764 | 9,095,546 | 639,558 | 60,498,933 | 3,771,160 |
| West | 2008 | 14,865 | 8596,469 | -499,077 | 57,574,088 | -2,924,845 |
| West | 2009 | 13,495 | 8,375,159 | -221,310 | 53,142,882 | -4,431,206 |
| West | 2010 | 11,433 | 7,938,285 | -436,874 | 45,749,708 | -7,393,174 |
| West | 2011 | 13,851 | 8,252,208 | 313,923 | 55,529,549 | 9,779,841 |
| West | 2012 | 13,817 | 8,537,995 | 285,787 | 54,880,626 | -648,923 |
| West | 2013 | 13,592 | 8,619,630 | 81,635 | 54,999,612 | 118,986 |
| West | 2014 | 11,468 | 8,099,302 | -520,328 | 47,271,404 | -7,728,208 |
| West | 2015 | 14,071 | 8,542,016 | 442,714 | 57,578,184 | 10,306,780 |
| West | 2016 | 14,568 | 9,562,761 | 1,020,745 | 61,183,703 | 3,605,519 |
| West | 2017 | 17,357 | 10,449,378 | 886,617 | 73,368,327 | 12,184,624 |
| West | 2018 | 23,312 | 11,818,482 | 1,369,104 | 99,170,351 | 25,802,024 |
| West | 2019 | 23,994 | 11,845,149 | 26,667 | 108,235,580 | 9,065,229 |
| West | 2020 | 26,474 | 12,666,417 | 821,268 | 117,412,653 | 9,177,073 |
| West | 2021 | 25,346 | 12,008,639 | -657,778 | 111,457,040 | -5,955,613 |
| West | 2022 | 22,198 | 11,407,061 | -601,578 | 98,839,937 | -12,617,103 |
| West | 2023 | 22,822 | 11,562,930 | 155,869 | 105,788,427 | 6,948,490 |

**Table S1:** Aggregated totals of fire days, population, and person-day exposure at the census-tract level, summarized by region and year.
